# Familial occipital neuralgia with *sporadic* nervus intermedius neuralgia (NIN)

**DOI:** 10.1007/s10194-011-0380-y

**Published:** 2011-09-04

**Authors:** Alex Alfieri, Christian Strauss

**Affiliations:** Department of Neurological Surgery, Martin Luther University Halle-Wittenberg, Halle, Germany

Sir,

We read with extreme interest the article of Wang and colleagues [1]. The observation of occipital neuralgia (ON) and nervus intermedius neuralgia (NIN) in every generation of one family suggests in fact a transmission of a hyperexcitability of both nerves. Currently, the most accredited theory is the mutation of the Nav1.7 sodium-channel with resulting familial inherited hyperexcitability [2, 3]. The paper is of interest because it describes the prospective consequences of the therapeutic modalities.

Nevertheless, we have one point of concern. In detail, only two of the five subjects presents NIN, associated with ON. The occipital nerve and the nervus intermedius have very different morphological, anatomical, embryological and functional characteristics. So the statement that both nerves may be impaired in the same family remains imprecise. The presented cases allow in fact concluding that the family presents a familial occipital neuralgia with *sporadic* NIN, presumably with XLD or AD inheritance.

## References

[1] Wang Y, Yu CY, Huang L, Riederer F, Ettlin D (2011). Familial neuralgia of occipital and intermedius nerves in a Chinese family. J Headache Pain.

[2] Fischer TZ, Waxman SG (2010). Familial pain syndromes from mutations of the NaV1.7 sodium channel. Ann N Y Acad Sci.

[3] Theile JW, Jarecki BW, Piekarz AD, Cummins TR (2011). Nav1.7 mutations associated with paroxysmal extreme pain disorder, but not erythromelalgia, enhance Navbeta4 peptide-mediated resurgent sodium currents. J Physiol.

